# Fermentation Performances and Aroma Contributions of Selected Non-*Saccharomyces* Yeasts for Cherry Wine Production

**DOI:** 10.3390/foods13152455

**Published:** 2024-08-03

**Authors:** Federico Bianchi, Michele Avesani, Marilinda Lorenzini, Giacomo Zapparoli, Barbara Simonato

**Affiliations:** 1 Department of Biotechnology, University of Verona, Strada le Grazie 15, 37134 Verona, Italy; federico.bianchi_02@univr.it (F.B.); michele.avesani@univr.it (M.A.); barbara.simonato@univr.it (B.S.); 2 Unione Italiana Vini, Viale del Lavoro 8, 37135 Verona, Italy; m.lorenzini@uiv.it

**Keywords:** cherry wine, yeast, *Hanseniaspora*, alcoholic beverage, volatile chemical compounds

## Abstract

This study evaluates the fermentation performances of non-*Saccharomyces* strains in fermenting cherry must from Italian cherries unsuitable for selling and not intended to be consumed fresh, and their effects on the chemical composition of the resulting wine. Fermentation trials in 100 and 500 mL of must were carried out to select 21 strains belonging to 11 non-*Saccharomyces* species. Cherry wines obtained by six select strains were chemically analyzed for fixed and volatile compounds. Quantitative data were statistically analyzed by agglomerative hierarchical clustering, partial least squared discriminant analysis, and principal component analysis. Wines revealed significant differences in their composition. Lactic acid and phenylethyl acetate levels were very high in wines produced by *Lachancea* and *Hanseniaspora*, respectively. Compared to *S. cerevisiae* wine, non-*Saccharomyces* wines had a lower content of fatty acid ethyl esters 4-vinyl guaiacol and 4-vinyl phenol. The multivariate analysis discriminated between wines, demonstrating the different contributions of each strain to aroma components. Specifically, all wines from non-*Saccharomyces* strains were kept strictly separate from the control wine. This study provided comprehensive characterization traits for non-conventional strains that enhance the aroma complexity of cherry-based wine. The use of these yeasts in cherry wine production appears promising. Further investigation is required to ascertain their suitability for larger-scale fermentation.

## 1. Introduction

Cherries are generally consumed fresh and are greatly appreciated by consumers for their sweet taste and the richness of several bioactive compounds [1]. However, in recent years, there has been an increase in defective fruits and significant preharvest losses due to excessive rainfall, which often causes fruit cracking. The defective cherries are unmarketable but can be upcycled through fermentation to formulate low-alcohol beverages (e.g., cherry wine) in the frame of sustainability.

Cherry wines are produced in many countries worldwide (e.g., China, the United States of America, India, and Europe). Cherries can also be used to produce other commercial beverages, like beer (i.e., kriek lambic) or cherry-based functional beverages obtained by lactic acid bacteria fermentation [2]. The consumer trend towards new and innovative foodstuffs [3] has boosted the demand for these beverages, like other fruit wines. Moreover, a UNEP report estimated that between 8 and 10% of global greenhouse emissions are associated with non-consumed food [4].

Non-*Saccharomyces* yeasts are considered important sources for producing fermented beverages due to their potentially positive effects on chemical and sensory quality [5,6]. Previous investigations have highlighted the impact of several species (e.g., *Hanseniaspora*, *Starmerella*, *Metschnikowia*, *Pichia*, *Zygosaccharomyces*, and *Lachancea*) on the aroma profile of beverages such as grape wine, cider, beer, and other fruit wines [7,8,9]. Specifically, these yeasts can increase the concentration of several molecules that activate odors (e.g., acetate esters, fatty acids, alcohols, and volatile phenols), and positively affect the sensory traits of these beverages. Moreover, the importance of the contribution of non-*Saccharomyces* yeasts in producing fermented beverages from berries rich in health-promoting bioactive compounds has been recognized [10].

Previous studies on the role of fermenting yeasts in cherry wine production have demonstrated the importance of their specific contribution to the aroma profile [11,12,13]. Sun et al. (2011) showed that *Saccharomyces cerevisiae* gave cherry wine different aromas and polyphenol profiles, depending on the strain used to ferment the must. The aptitude of other yeast species, such as *Torulaspora delbrueckii* and *Metschnikowia pulcherrima*, to ferment cherry must and confer cherry wine’s peculiar organoleptic quality has been evaluated [12,13]. Mixed fermentations with non-*Saccharomyces* yeasts and *S. cerevisiae* led to a significant variation in the chemical and sensory levels when a yeast combination was used as a starter for cherry wine production [12]. These promising data suggest that evaluating the potential of other yeast species in producing this beverage would be profitable. 

This study aimed to evaluate the ability of different non-*Saccharomyces* strains to ferment cherry must and their potential contribution to producing aroma molecules in the resulting wine. A preliminary screening of several strains based on their fermentation capacity was conducted to select those suitable for fermentation of must from Italian cherries not intended for fresh consumption. Chemical analysis was performed to investigate variations in the aroma composition of fermented beverages, depending on the strain used. The evaluation of the suitability of yeasts as starters in the fermentation process to produce cherry-based wine is discussed below.

## 2. Materials and Methods

### 2.1. Strains and Culture Conditions

A total of 21 strains were analyzed in this study (Table S1). Eighteen were isolated and identified in previous investigations [14,15]. Three strains, Lf2, Wa847, and Zb23, isolated from olive oil, wine, and vinegar, respectively, were identified in this study through the sequence analysis of the D1/D2 domain of the 26S rRNA gene as previously described [14]. The commercial strain *S. cerevisiae* EC1118^®^ (Lallemand Inc., Montréal, QC, Canada) was used as control. All strains were grown on YPD (10 g/L yeast extract, 20 g/L peptone, 20 g/L glucose) at 25 °C and maintained in a slant of the same medium with 15 g/L agar at 4 °C.

### 2.2. Must Preparation for Fermentation Assays

Figure S1 shows the experimental design of this study. Assays were carried out in a grape must as a natural substrate for preliminary screening of the fermentation ability of yeasts, and then the selected strains were assayed in cherry must. Grape must (sugars 17.6 °Brix, pH 3.23, titratable acidity 6.58 g/L as tartaric acid) was obtained by manually crushing destemmed grapes of Garganega variety, pasteurized at 80 °C for 5 min. 

Cherry must was obtained from cherries of Italian local varieties, mainly Mora di Cazzano, that were not tradable for fresh consumption due to their low quality and were used for fermentation assays. Two bulk quantities of cherries, retrieved at two different times (April and May 2023) during the harvested season, were used to prepare musts A and B by crushing the cherries with a juice extractor. Must A (sugars 14.1 °Brix, pH 3.97, malic acid 9.1 g/L) was used for the first trial, and must B (sugars 12.6 °Brix, pH 3.59, malic acid 8.6 g/L) was used for a subsequent fermentation trial to evaluate the contribution of selected yeasts to aroma production. Must A was pasteurized at 80 °C for 5 min, while must B, used in the trial for aroma analysis of the fermented product, was not thermally treated to avoid partial alteration of aroma precursors.

### 2.3. Fermentation Trials, Strain Inoculation and Microbial Analysis

A total of 21 strains were assayed in the preliminary fermentation trial on grape must performed in a volume of 100 mL of must in a 200 mL flask. Ten selected strains were assayed in cherry must A in a volume of 100 mL, contained in a 200 mL flask, while those with cherry must B had a volume of 350 mL in a 500 mL bottle. Each flask or bottle was inoculated by the strain from a pure culture grown in YPD for 3 days at a cellular density of approximately 10^6^ cells/mL. Flasks or bottles were incubated at 25 °C, and the fermentation course was analyzed by weight loss every 1–2 days. The CO_2_ production was stoichiometrically determined by measuring the weight loss and fermentation was considered terminated when weight loss ceased [16]. Three independent trials of each strain were performed in all fermentation assays.

### 2.4. Microbial Counts

Microbial analysis was performed by sampling 5 mL from cherry musts, and by plating diluted aliquots on WL agar, without and with cycloheximide (Merck KGaA, Darmstadt, Germany) (10 mg/L) and MRS agar (Condalab, Madrid, Spain) with cycloheximide (10 mg/L) to verify the presence of indigenous microorganisms before the inoculation. Moreover, this analysis was carried out at the end of fermentation of must B trials to ascertain that the inoculated strain colonized the must and performed the fermentation. Aliquots of diluted must and/or wine collected from each trial were plated on WL medium and, after 3 days of incubation at 25 °C, colonies and cells were analyzed for their morphology. Some representative yeast colonies were isolated and molecularly identified by sequencing the D1/D2 region, described above. 

### 2.5. Chemical Analysis of Must and Wine

Sugars contained in grape and cherry musts were measured using a HI 96801 digital refractometer (Hanna Instrument, Woonsocket, RI, USA). Titratable acidity, expressed as tartaric acid, was determined in grape must by the Office International Organization of Vine and Wine (OIV) method (OIV-MA-AS313-01, 2021). In cherry wine, glucose and fructose, organic acids (acetic acid, malic acid, lactic acid, gluconic acid), and glycerol were determined using a Hyperlab wine analyzer with enzymatic kits (Steroglass Srl, Perugia, Italy) (OIV-MA-AS311-02, 2021; OIV-MA-AS313-27, 2021; OIV-MA-AS313-26, 2021; OIV-MA-AS313-25, 2021; OIV-MA-AS313-28, 2021). Ethanol was determined by NIR spectrometry using an Alcolyzer (Anton Paar GmbH, Graz, Austria). Total polyphenols (expressed as gallic acid) were measured according to the Folin–Ciocalteu colorimetric method (OIV-MA-AS2-10, 2021), and color intensity was determined spectrally by measuring absorbance at 420, 520, and 620 nm (OIV-MA-AS2-11, 2021).

Volatile compounds of cherry wine were extracted by solid phase extraction (SPE) using 1 g ENV^+^ cartridges (Isolute, IST Ltd., Mid Glamorgan, UK) and performed on an automated solid phase extraction instrument (Aspec XL Gilson Inc., Middleton, USA). Diluted samples of wine with 1-heptanol as internal standard (500 μg/L) were loaded onto the SPE cartridge. Then, the cartridge was washed with water (10 mL) and the volatile compounds were eluted with dichloromethane (9 mL). The collected solution was dried with sodium sulphate and concentrated to about 400 μL under a gentle nitrogen flow.

GC-MS analysis was conducted using an Agilent 6890N gas chromatograph equipped with an Agilent 5978B mass selective detector (Agilent Technologies, Milan, Italy). The SPE extraction was analyzed on an HP-WAX Bonded PEG fused silica capillary column (60 m × 0.320 mm i.d., 0.25 μm film thickness; Agilent Technologies). Each extraction was injected with splitless. The oven temperature was kept at 50 °C for 4 min, then raised to 240 °C at a rate of 4 °C/min, and held at 240 °C for 16 min. The mass spectrometer operated in electron ionization (EI) at 70 eV, and the ion source temperature was set at 230 °C.

Compound identifications were carried out by comparison of the retention time obtained for the same samples using commercial pure reference standards (Merck KGaA, Darmstadt, Germany) injected under the same conditions and comparing the mass spectra with those given in the spectra database of the National Institute of Standard and Technology (NIST), considering above 70% similarity. The tentative identification of other volatile compounds was performed by comparison of the retention index with that reported in the literature and by comparison of MS fragmentation with those reported in the NIST mass spectral database library. Standard curve concentration and compounds were quantified based on the ratio of the molecule’s peak area relative to the internal standard’s peak area to determine the molecules’ concentration. 

Odor activity values (OAVs) were calculated as the ratio between the measured concentration of a substance in the wine and its odor threshold, when available.

### 2.6. Statistical Analysis

The fermentation parameters (CO_2_ production amounts and rates) measured in non-*Saccharomyces* strains were compared with those obtained by *S. cerevisiae* EC1118^®^, and significant differences were calculated by the t-test using Excel software (Version 2406 Build 16.0.17726.20078, Microsoft Corporation, Redmond, WA, USA).

Variance analysis (ANOVA) was used to analyze each chemical compound of cherry-based wines, presented as mean and standard deviation from three independent trials, and significant differences between samples were determined by applying Tukey’s multiple comparison test. 

Agglomerative hierarchical clustering (AHC) was performed using Euclidean distances to calculate dissimilarities and Ward’s method for clustering. The heat map dendrograms made with AHC of aroma components of cherry-based wines were created, displaying the magnitude of each compound value with color from white (low) to red (high) depending on its normalized concentration. 

Partial least squared discriminant analysis (PLS-DA) was carried out on the normalized value of aroma compounds to determine potential volatile aroma markers. The dataset was cross-validated randomly and following the Jack-knife method of regression. The prediction was conducted on a different subset of data, and the confidence interval was set at 95%. Thus, the goodness of fit parameter for both variables (R^2^X and R^2^Y) and the goodness of prediction parameter (Q^2^) were derived. The most influential aroma markers that were responsible for the discrimination of fermented cherry-based wine juice were determined by the variable importance in projection (VIP) scores calculated through the PLS-DA models.

Principal component analysis (PCA) was performed using values of all physical-chemical components of cherry-based wine produced by each yeast strain. Data were automatically scaled by the program (Person as PCA type).

ANOVA, heat maps, AHC, PLS-DA, and PCA were generated and conducted on Excel software (Version 2406 Build 16.0.17726.20078, Microsoft Corporation) using XLSTAT package software (XLSTAT 2023.2.1413) (Addinsoft SARL, Paris, France) on the normalized data of different cherry-based wine compounds.

## 3. Results

### 3.1. Preliminary Screening on Fermentation Capacity

Table 1 reports the CO_2_ production parameters of 21 strains and *S. cerevisiae* EC1118^®^ (control yeast), measured during grape must fermentation. The total amount of CO_2_ production ranged from 3.9 to 7.6% (*w*/*v*), and 14 out of 21 strains produced at least 7.3% (*w*/*v*), a value not significantly different from that of control yeast (7.6% *w*/*v*). The CO_2_ produced after three days ranged from 0 to 3.7% (*w*/*v*), while that of control yeast was significantly much higher (6.8% *w*/*v*). The average daily rate of CO_2_ production was similar in all strains, ranging from 0.3 to 0.4% (*w*/*v*) per day (except *W. anomalus* Wa847 and *H. meyeri* Y1A). In contrast, the maximum daily rate varied from 0.4 to 2.3 (*w*/*v*), significantly lower than that of control yeast (3.3% *w*/*v* per day).

According to these results, some strains were selected for further assays on cherry must fermentation. The main selection criteria were the total amount and maximum daily rate of CO_2_ produced and the species to which the strain belongs. The selection of strains to use on further assays was carried out among those able to produce more than total CO_2_ of 6.0% (*w*/*v*), an arbitrary level considered enough to completely ferment a cherry must with a minimum reducing sugar content of 100 g/L. Then, 10 strains were selected for fermenting cherry musts, and only some representative strains of the same species were considered.

### 3.2. Fermentation of Cherry Must

**Figure 1:**
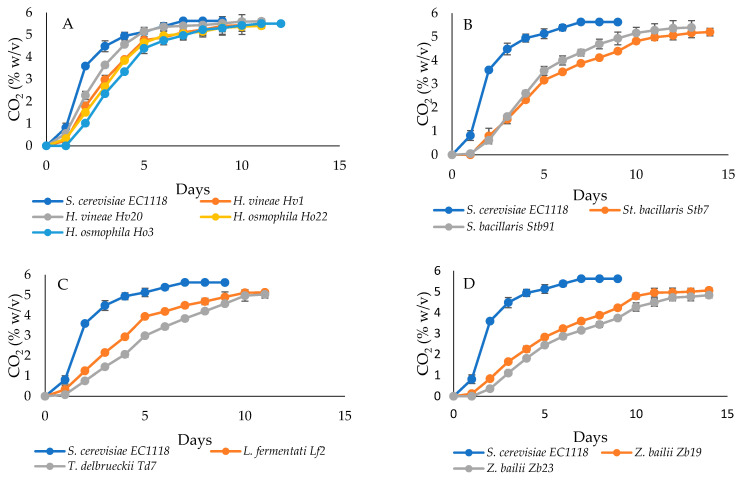
Kinetic of CO_2_ production (% *w*/*v*) during the fermentation of pasteurized cherry must A by 10 selected yeasts and *S. cerevisiae* EC1118^®^. (**A**) Kinetics of CO_2_ production of strains *S. cerevisiae* EC1118, *H. vineae* Hv1, *H. vineae* Hv20, *H. osmophila* Ho22, and *H. osmophila* Ho3; (**B**) Kinetics of CO_2_ production of strains *St. bacillaris* Stb7, and *S. bacillaris* Stb91; (**C**) Kinetics of CO_2_ production of strains *L. fermentati* Lf2, and *T. delbrueckii* Td7; (**D**) Kinetics of CO_2_ production of strains *Z. bailii* Zb19, and *Z. bailii* Zb23.

The 10 selected yeasts were assayed in a first trial on the pasteurized must of cherry (must A). *Hanseniaspora* strains showed CO_2_ production kinetics quite similar to that of *S. cerevisiae* EC1118^®^, although they had the start of fermentation postponed (Figure 1). The kinetics of the other six strains were more different for total CO_2_ produced, slightly less than control yeast, and delayed at the start. Based on these results, six strains (*H. osmophila* Ho3, *H. vineae* Hv20, *L. fermentati* Lf2 *St. bacillaris* Stb91, *T. delbrueckii* Td7, and *Z. bailii* Zb19), representing each species, were further selected for fermentation trials in cherry must B (non-pasteurized). Strains colonized must B and dominated the fermentation in all trials according to the results of the microbiological analysis. The kinetics of CO_2_ production were similar to those observed in the trial in cherry must A (Figure S2). Wines obtained by each strain (labeled with HO, HV, LF, STB, TD, and ZB) were chemically analyzed.

### 3.3. Chemical Analysis of Wines

The most relevant differences among wines’ primary composition were pH, glucose and fructose content, ethanol, total acidity, lactic acid, glycerol, and total polyphenols (Table 2). In particular, wine ZB had the highest glucose and fructose concentration and the lowest ethanol content (35 g/L and 6.1% *v*/*v*, respectively). Wine LF had a low pH (3.3) due to the high lactic acid concentration (4.4 g/L). Wines fermented by *L. fermentati* Lf2, *St. bacillaris* Stb91, and *Z. bailii* Zb19 had a content of glycerol 1.8 g/L or more than the other four wines. Wine ZB was characterized by a total polyphenol content of at least 1000 mg/L higher than the other wines.

The quantification of several molecules important to the aroma profile of cherry-based wine revealed substantial differences among non-*Saccharomyces* strains and between these and the control strain *S. cerevisiae* EC1118^®^ (Table 2). Several compounds were detected above their respective thresholds (Table S3). For instance, TD, HO, HV, and ZB wines had higher OAV for eugenol than the control wine; thus, it is expected to present a more intense clove and spice aroma. HV and HO wines present more intense rose/floral and cinnamon/balsamic scents due to phenylethyl acetate and ethyl cinnamate, respectively. The total amount of acetate esters, fatty acid ethyl esters, and fatty acids and their ratio considerably changed among wines (Figure S3). The ratio of fatty acid ethyl esters and acetate esters varied greatly from 0.3 (wine HV) to 83 (wine STB). In contrast, the variation of the ratio of fatty acid ethyl esters and fatty acids was smaller, ranging from 0.4 (wine HV) to 3.5 (wine STB). Concerning acetate esters, isoamyl acetate was lower in all non-*Saccharomyces* wines than in the control wine (e.g., its ratio of control wine and wine STB was 210). In comparison, phenylethyl acetate was higher in wines HV, HO, and LF and lower in wines TD, STB, and ZB. The behavior of these two compounds, detected above their respective odor thresholds, determined the variation in related OAVs. The isoamyl acetate OAV in the control wine was 31.5, while it ranged between 0.2 and 5.2 in all non-*Saccharomyces* wines.

Most of the reduction in the total amount of fatty acid ethyl esters observed in all wines with respect to the control was due to ethyl-4 hydroxybutyrate, which was the predominant ester in almost all wines. However, the highest reduction rate between the control wine and non-*Saccharomyces* wines was observed in ethyl butyrate, ethyl hexanoate, and ethyl octanoate content. In particular, the ethyl hexanoate ratio of the control wine and other wines was up to 177 (wine STB), and this reduced its OAV from 20.3 in the control wine to 0.1. Conversely, ethyl decanoate varied differently among non-*Saccharomyces* wines, and only in wine HV was this molecule above its odor threshold (OAV = 1.4). Regarding other esters, the substantial increase in ethyl lactate in wine LF (1100% with respect to the control wine) is worth mentioning, while the ethyl cinnamate was above its odor threshold in all wines.

The reduction in fatty acid concentration in non-*Saccharomyces* wine with respect to the control wine was mainly due to hexanoic acid, octanoic acid, and decanoic acid (the ratio of these acids in the control wine and wine STB was 28, 84, and 23, respectively). Decanoic acid increased only in wine HV (47%). The behaviors of these three fatty acids had a relevant impact on their respective OAVs. Dodecanoic acid was exceptionally high in wine HO, with a ratio of 5.5 with the control wine.

Concerning alcohols, it is worth mentioning the variation in 2-phenylethanol, which decreased up to 42% (wine HO) and increased up to 35% (wine ZB) with respect to the control wine, and the substantial reduction in 3-methylthio-1-propanol in all non-*Saccharomyces* wines, of up to −93% in wine TD.

A very low content of 4-vinyl guaiacol and 4-vinyl phenol was detected in all non-*Saccharomyces* wines. Their control wine and wine HO ratio was 31 and 300, respectively. These two molecules were above their respective odor thresholds only in control wine, except 4-vinlyguaiacol, which had OAV = 1.4 in wine TD.

Lactones considerably varied among wines. γ-Butyrolactone, the predominant molecule of this chemical class, increased up to 173% (wine LF), and the content of sherry lactone 2 increased by up to 235% (wine TD) and that of ethyl pyroglutamate by up to 336% (wine LF) with respect to control wine.

Phenylacetaldehyde, above its odor threshold in all wines, varied differently among wines, increasing up to 113% (wine TD) and decreasing up to 40% (wine LF). Benzaldehyde was found to be lower in non-*Saccharomyces* wines, up to −50% (wine STB) with respect to the control wine.

### 3.4. Statistical Analysis of Wines

**Figure 2:**
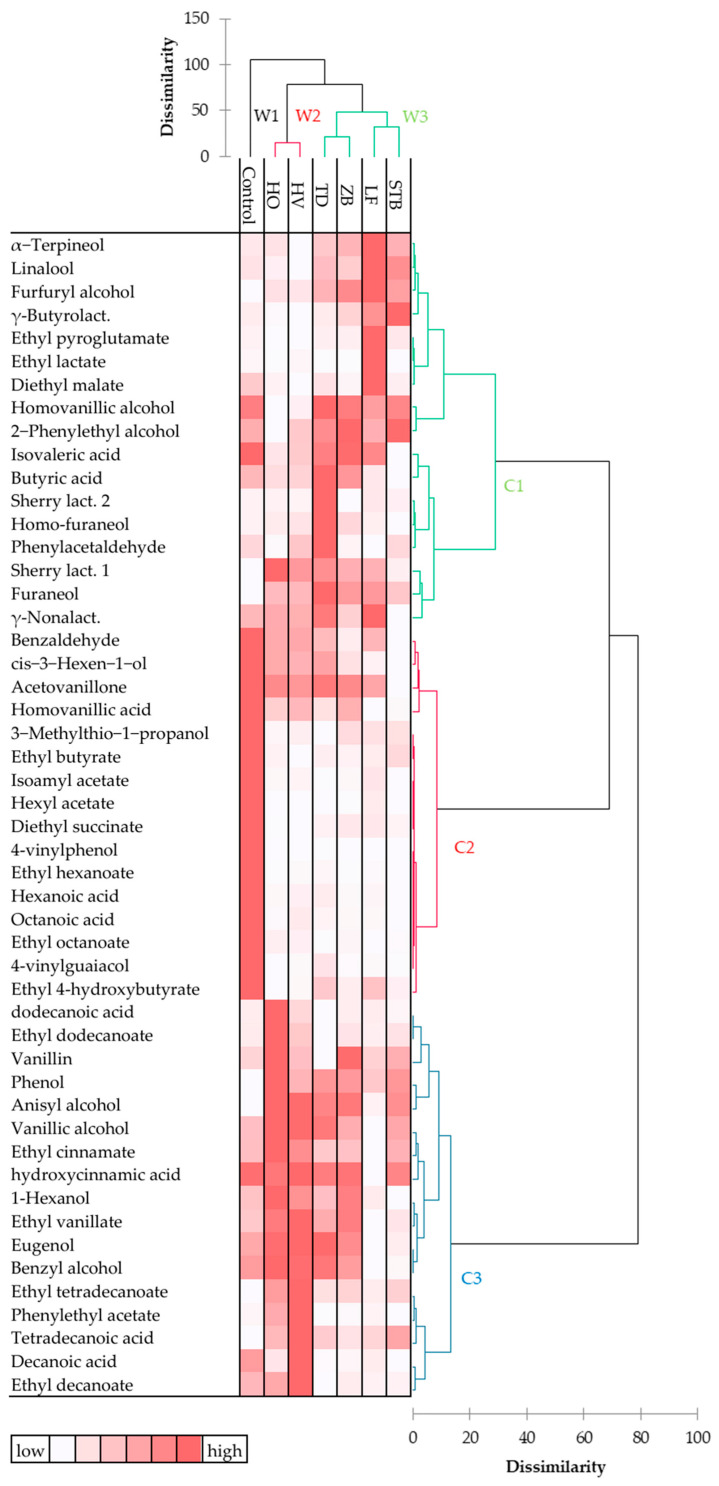
Heat map with dendrograms made with AHC corresponding to aroma compounds of cherry-based wines produced by *S. cerevisiae* EC1118^®^ (control), *T. delbrueckii* Td7 (TD), *H. vineae* Hv20 (HV), *H. osmophila* Ho3 (HO), *L. fermentati* Lf2 (LF), *St. bacillaris* Stb91 (STB), and *Z. bailii* Zb19 (ZB). The relative content of each molecule is illustrated through a color scale from white (minimum) to dark red (maximum).

The composition of cherry wines was statistically analyzed to highlight the relationship between the aroma profiles and the fermenting yeasts. The Hartigan index obtained by HCA indicated three clusters for cherry-based wines and aroma compounds (Table S3). The heat map displays wines produced by non-*Saccharomyces* yeasts grouped into two main clusters, W2 (wines HO and HV) and W3 (wines TD, ZB, LF, and STB), while wine from *S. cerevisiae* EC1118^®^ was well separated (W1) (Figure 2). Aroma compounds were clustered into three groups (C1, C2, and C3). Cluster C2 had higher homogeneity than the other two, C1 and C3, due to the high quantity of all compounds (in particular, fatty acid ethyl esters, fatty acids, and vinylphenols) detected in the control wine.

**Figure 3:**
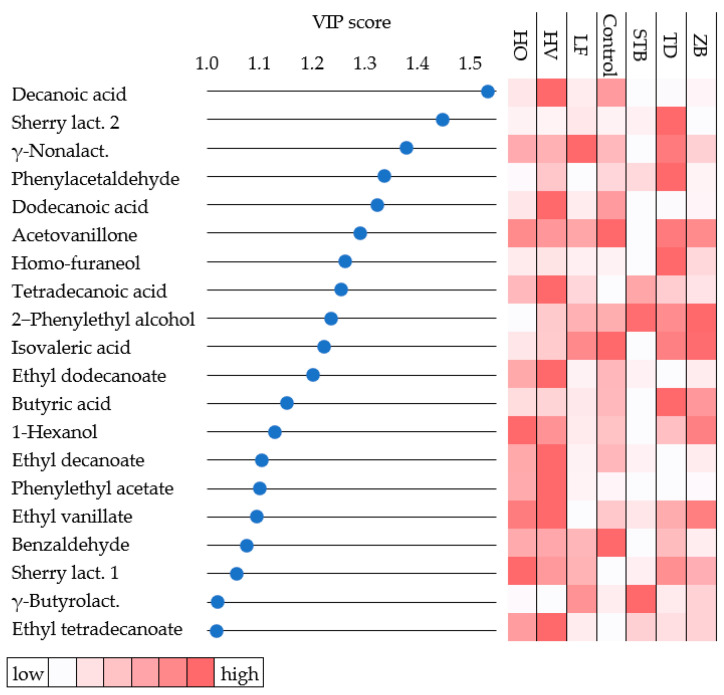
VIP score (>1) and relative content (color scale from white as minimum to red as maximum) of aroma compounds of cherry-based wines produced by *S. cerevisiae* EC1118^®^ (control), *T. delbrueckii* Td7 (TD), *H. vineae* Hv20 (HV), *H. osmophila* Ho3 (HO), *L. fermentati* Lf2 (LF), *St. bacillaris* Stb91 (STB), and *Z. bailii* Zb19 (ZB) obtained by PLS-DA analysis.

Supervised PLS-DA was performed to identify potential aroma compounds to discriminate fermented cherry-based wine by different strains. The R2X, R2Y, and Q2 values were 0.95, 0.97, and 0.89, respectively, indicating that the built model has a satisfying goodness of prediction (Q2 > 0.5). The list of VIP scores, calculated through the PLS-DA model, accounted for 20 compounds with a score greater than 1, indicating that these variables were the most important in contributing to the model (Figure 3). Among these 20 compounds, fatty acids and lactones accounted for nine molecules, of which decanoic acid, sherry lactone 2, and γ-Nonalactone had the highest score (1.53, 1.45, and 1.38, respectively). The control wine was separated from non-*Saccharomyces* wines in the PLS-DA plot. Wines ZB and TD were not visibly distinguishable, while wines LF and STB were clearly distinct (Figure S4).

PCA, carried out using fixed and volatile compounds of each wine, clearly revealed the effects of yeast on the chemical composition of cherry-based wine (Figure S5). This analysis, which explained a cumulative variability of 60% by the first two factors (F1 = 30.7% and F2 = 28.9%), confirmed the good separation of non-*Saccharomyces* wine samples from the control one, as well as wines TD and ZB, even though they were close. A total of 23 compounds, including 9 of them detected above their respective thresholds in at least one wine (e.g., isoamyl acetate, ethyl butyrate, hexanoic acid, and 4-vinyl guaiacol), showed a significant loading score (<−0.800 or >0.800) in F1 and/or F2 (Table S4).

## 4. Discussion

The fermentation performance of different strains belonging to the species in grape must used in this study is widely documented [17]. The result of the fermentation trial of *H. osmophila* strains was consistent with Granchi et al. (2002), and another in *H. vineae* strains was in accordance with the findings of Del Fresno et al. (2021) [9,18]. The fermentation capacity of *St. bacillaris* and *Z. bailii* strains confirmed that these species are suitable to be used in mixed fermentation for alcoholic beverages [8,19]. The better performance of *L. fermentati* Lf2 compared to *L. lanzarotensis* strains concurred with the findings of Porter, Divol, and Setati (2019) [20]. Trials on cherry musts demonstrated that the selected strains can ferment cherry musts, and their sugar/ethanol conversion rate is compatible with cherry wine production from musts with low and medium sugar concentrations (less than 120 g/L). For cherry musts that are richer in fermentable sugars, including with the addition of sucrose or blending other fruit musts (e.g., grape), association with *S. cerevisiae* may be necessary to complete the fermentation. The average ethanol content of commercial cherry wine ranges from 8 to 12% *v*/*v*, and rarely up to 18% *v*/*v*, as also reported by Xiao et al. (2017) and Nui et al. (2019) [21,22]. Among non-*Saccharomyces*, only a few species, including *T. delbrueckii*, *St. bacillaris*, and *Hanseniaspora* spp., have a high capacity in converting sugars to ethanol at levels above 10% *v/v* [23].

As regards the chemical composition of cherry wines, the acidification observed in wines obtained by *L. fermentati* Lf2 was due to the production of lactic acid during alcoholic fermentation, a metabolic trait that has been thoroughly investigated, especially in *L. thermotolerans* [24]. Bellut et al. (2020) evaluated the use of *L. fermentati* to produce low-alcohol beer and reported that the lactic acid production was strain-dependent [7]. Significant variations in total polyphenol content observed among cherry-based wines were consistent with the different capacities of yeasts to increase some phenolic compounds (e.g., protocatechuic acid, p-coumaric acid, ferulic acid, and p-hydroxybenzoic acid) in grape wines, as described by [25]. The high content of total polyphenols in wine ZB could be attributed to the greater efficiency of *Z. bailii* in synthesizing polyphenols via the shikimate pathway. However, little information is available on this metabolic pathway in non-*Saccharomyces* yeasts [26].

With regard to the different ratios of the content of essential molecules responsible for fruity scents, such as acetate esters, ethyl fatty acid ethyl esters, and fatty acids, the role played by *Hanseniaspora* in increasing acetate esters is recognized [27]. Recently, Seixas, Santos, Vasconcelos, Mira, and Mendes-Ferreira (2023) pointed out that the over-production of acetate esters by *Hanseniaspora* spp. is accompanied by a higher expression of the alcohol-acetyl transferase gene family than *S. cerevisiae*. Moreover, greater duplication and the absence of genes involved in producing these molecules were identified in the genome of *H. vineae* compared to *S. cerevisiae* [28]. These observations are consistent with the large quantity of phenyethyl acetate in cherry-based wines produced by *H. vineae* Hv20 and *H. osmophila* Ho3. In these wines, this OA compound was found at concentrations many times (up to 83 times) higher than in commercial Chinese cherry wines [21,22]. The considerable impact that *Hanseniaspora* spp. has on the aroma profile of cherry wines appears evident since the floral and fruit scents are important sensory descriptors of this type of beverage [21,22]. Conversely, data on wines TD, STB, and ZB, which were poor in terms of phenylethyl acetate, isoamyl acetate, and fatty acid ethyl esters, indicate a reduced intensity of these sensory notes. The low content of fatty acid ethyl esters, in particular ethyl butyrate, ethyl hexanoate, and ethyl octanoate, in cherry wine fermented by non-*Saccharomyces* strains was consistent with data observed in grape or fruit wines fermented by various strains belonging to the same species used in this study, such as *T. delbrueckii*, *H. vineae*, *St. bacillaris*, and *Z. bailii*, when compared with *S. cerevisiae* [10,12,15,29]. Since the contribution of ethyl butyrate, ethyl octanoate, and ethyl decanoate to the cherry wine aroma is very important [21,22], a low amount quantity of these OA compounds may significantly affect the sensory properties of wine. These differences could be associated with the lower biosynthesis capacity of these esters compared to *Saccharomyces*. Giorello et al. (2019) reported that the absence of the ethanol O-acyltransferases (EEB1) gene in *H. vineae* reduces the production of ethyl esters and fatty acids [28]. Transcriptome analysis of *T. delbrueckii* and *S. cerevisiae* revealed essential differences in the expression level of key enzymes involved in producing acetates, ethyl esters, and fatty acids during alcoholic fermentation [30]. Significant variations observed not only in the content of esters and fatty acids, but also in alcohols in the cherry wines of this study, could be related to the specific metabolic traits of each strain used in the fermentation. Studies comparing *Hanseniaspora* yeasts to *S. cerevisiae* found substantial differences in the production of aroma molecules, including alcohols such as 3-methylthio-1-propanol and 2-phenylethanol, which were found at significantly different concentrations in the cherry wines in this study [28,31].

The variations in other molecules, such as ethyl lactate, vinylphenols, and lactones, could also be attributable to the distinct metabolic profiles of each strain. The very high content of ethyl lactate detected in wine LF is due to the significant lactic acid production of *L. fermentati*, as cited above. The low concentration of 4-vinylphenol and 4-vinylguaiaol in all cherry wines suggests that the ability to decarboxylate aromatic carboxylic acids, like ferulic acid and p-coumaric, was reduced or absent in non-*Saccharomyces* strains. These results are consistent with our previous investigation into ciders, in which the content of 4-vinylguaiacol was much lower in those fermented by strains of *T. delbrueckii*, *H. osmophila*, *St. bacillaris*, and *Z. bailii* than in cider obtained with *S. cerevisiae* [15]. Mukai et al. (2014) reported that some *S. cerevisiae* strains, including strain EC1118^®^, had ferulic acid decarboxylation ability, although this was absent in other strains [32]. This evidence may explain the data of Nui et al. (2011) [33], reporting that cherry wines were found to contain 4-vinyl guaiacol at a level of between 0 to 1.1 mg/L. Further investigation is required to understand the role of non-*Saccharomyces* yeasts in synthesizing these volatile phenols, given their importance in affecting the quality of cherry wine.

Among lactones, the relatively high concentration of ethyl pyroglutamate and γ-butyrolactone in wine LB could be associated with the specific traits of *Lachancea* species. Vaquero et al. (2021) revealed an increase in γ-butyrolactone in wine obtained by sequential fermentation of *L. thermotolerans* compared to wine produced only by *S. cerevisiae* [34]. However, these results could also be related to strain-specific metabolic differences rather than species-specific ones. The level of γ-butyrolactone in wine STB suggests that *St. bacillaris* Stb91 is a highly prodigious producer of this molecule, in line with Russo et al. (2020), which showed substantial differences in wines produced by different strains of this species [35].

Multivariate statistical techniques, used to analyze the chemical composition of wines, well supported the discrimination of wines. The clustering of wines provided by HCA, PLS-DA, and PCA plots highlighted the distance of *S. cerevisiae* from non-*Saccharomyces* wines, demonstrating a similar discriminatory power, including the differences observed among non-*Saccharomyces* wines. Moreover, these three techniques extracted specific information on the most discriminative features of each wine, which is useful for interpreting the role of the related fermenting yeast. The clustering of volatile compounds generated by HCA evidenced the molecules that contributed most to the discrimination of wines. Interestingly, HCA cluster C2 included molecules that have a relatively high content in *S. cerevisiae* wine and significant PCA loading factors (except acetovanillone). On the other hand, HCA clusters C1 and C3, with high content in W2 and W1 cluster wines, included most of the molecules with high PLS-DA VIP scores. These results confirm the importance of using different multivariate analysis techniques to recover the most information from data provided by volatile compound analysis of alcoholic beverages [36].

## 5. Conclusions

This study highlights the role of non-*Saccharomyces* yeasts in producing cherry-based wines and evaluated their fermentation performance and impact on the aroma profiles of experimental fermented beverages. In particular, assessing yeast species, such as *H. vineae*, *H. osmophilia*, *L. fermentati*, *St. bacillaris,* and *Z. bailii*, is an innovation in cherry wine production. The multivariate analysis well confirmed each species’ different contributions to cherry-based wine’s chemical quality throughout their discrimination, not only from *S. cerevisiae*, but from each of them. The complexity of aroma profiles can be explained by their specific metabolic traits, which have been thoroughly investigated in grape wine fermentation, especially in some of these species. However, the composition of cherry must significantly differs from that of grapes, and the effects on yeast metabolisms and the consequent ability to convert precursors to metabolites may also be very different. In addition, the significant variability in the composition of cherry cultivars may noticeably increase the multiplicity of aroma and flavor production, which may be obtained using non-*Saccharomyces* yeasts. Several aspects of OA molecule production characteristic of cherry wine obtained by these yeasts have yet to be clarified. Nevertheless, further experimentation is necessary to evaluate the scale-up of cherry wine production with non-*Saccharomyces* yeasts. *Torulaspora delbrueckii, H. vineae*, and *H. osmophila* represent the most promising strains regarding fermentation ability and aroma development.

## Figures and Tables

**Table 1:** Parameters of CO_2_ production (% *w*/*v*, total amount at the end of fermentation and after 3 days, average and maximum daily rate) of 21 strains and *S. cerevisiae* EC1118^®^ (control) obtained during the grape must fermentation.

Strains	Total Amount	Amount after 3 d	Average Daily Rate	Maximum Daily Rate
*S. cerevisiae* EC1118^®^	7.6 ± 0.2	6.8 ± 0	0.4 ± 0	3.3 ± 0.1
*St. bacillaris* Stb7	7.6 ± 0.1	0.9 ± 0.1 **	0.4 ± 0.0	1.2 ± 0.1 **
*H. osmophila* Ho22	7.5 ± 0.2	0 ± 0 **	0.4 ± 0.0	1.3 ± 0.1 **
*St. bacillaris* Stb142	7.5 ± 0.2	0 ± 0 **	0.3 ± 0	1.2 ± 0.1 **
*St. bacillaris* Stb3	7.5 ± 0	0 ± 0 **	0.3 ± 0	1.3 ± 0 **
*H. vineae* Hv1	7.4 ± 0	3.7 ± 0.2 **	0.4 ± 0	2.3 ± 0.1 **
*H. vineae* Hv20	7.3 ± 0.2	1.7 ± 0 **	0.4 ± 0.2	1.6 ± 0.1 **
*St. bacillaris* Stb91	7.3 ± 0.1	2.1 ± 0 **	0.4 ± 0	1.5 ± 0.1 **
*T. delbrueckii* Td7	7.3 ± 0.1	0.0 ± 0 **	0.3 ± 0	1.2 ± 0 **
*Z. bailii* Zb19	7.3 ± 0.1	1.8 ± 0 **	0.3 ± 0	1.2 ±0.1 **
*Z. bailii* Zb23	7.3 ± 0	0.9 ± 0.1 **	0.3 ± 0	0.8 ± 0.1 **
*H. osmophila* Ho3	7.2 ± 0.1 *	1.7 ± 0.1 **	0.4 ± 0.1	1.3 ± 0 **
*H. vineae* Hv45	7.1 ± 0 *	0.1 ± 0 **	0.4 ± 0	1.9 ± 0.1 **
*St. bacillaris* Stb34	7.1 ± 0.1 *	0 ± 0 **	0.3 ± 0	1.5 ± 0.1 **
*Z. bailii* Zb17	7.1 ± 0.1 *	1.2 ± 0.2 **	0.3 ± 0	1.0 ± 0.1 **
*L. fermentati* Lf2	6.7 ± 0.1 **	2.6 ± 0.1 **	0.3 ± 0	1.5 ± 0.1 **
*L. lanzarotensis* Ll5	6.0 ± 0.2**	0.1 ± 0.1 **	0.3 ± 0.1	1.2 ± 0.1 **
*H. pseudoguilliermondii* YR6	5.8 ± 0.2 **	0 ± 0 **	0.3 ± 0 *	0.9 ± 0.1 **
*H. valbiensis* Y2D	5.6 ± 0.1 **	0.1 ± 0 **	0.3 ± 0	1.1 ± 0 **
*L. lanzarotensis* Ll7	5.4 ± 0.1 **	2.6 ± 0.1 **	0.4 ± 0	1.5 ± 0.2 **
*W. anomalus* Wa847	5.0 ± 0.2 **	0.1 ± 0.1 **	0.2 ± 0.1	0.4 ± 0 **
*H. meyeri* Y1A	3.9 ± 0.1 **	1.4 ± 0 **	0.2 ± 0 *	1.0 ± 0.2 **

**, *, within the column significantly different to *S. cerevisiae* EC1118^®^ at *p* < 0.01 and 0.05, respectively.

**Table 2:** Composition of cherry-based wines produced by *S. cerevisiae* EC1118^®^ (control), *T. delbrueckii* Td7 (TD), *H. vineae* Hv20 (HV), *H. osmophila* Ho3 (HO), *L. fermentati* Lf2 (LF), *St. bacillaris* Stb91 (STB), and *Z. bailii* Zb19 (ZB). Values are mean with standard deviation of three independent trials.

	RI ^1^	ID ^2^	Control	TD	HV	HO	LF	STB	ZB
Primary compounds									
pH			3.7 ± 0.0 cd	3.6 ± 0.0 d	3.8 ± 0.0 a	3.8 ± 0.0 b	3.3 ± 0.0 e	3.7 ± 0.0 c	3.9 ± 0.1 a
Glucose and fructose (g/L)			2.7 ± 0.1 e	10.5 ± 1.0 c	4.7 ± 0.5 de	6.8 ± 1.7 cd	6.0 ± 0.4 de	19.1 ± 2.7 b	35.9 ± 1.8 a
Ethanol (% *v/v*)			8.0 ± 0.0 a	7.6 ± 0.1 b	8.1 ± 0.0 a	8.0 ± 0.1 a	7.4 ± 0.0 bc	7.1 ± 0.1 c	6.1 ± 0.4 d
Acetic acid (g/L)			0.2 ± 0.0 c	0.3 ± 0.0 c	0.4 ± 0.0 ab	0.3 ± 0.0 bc	0.5 ± 0.0 a	0.5 ± 0.0 a	0.5 ± 0.1 a
Malic acid (g/L)			8.4 ± 0.0 a	8.3 ± 0.2 a	6.7 ± 0.2 c	7.2 ± 0.0 bc	7.6 ± 0.0 b	7.0 ± 0.2 c	8.2 ± 0.3 a
Lactic acid (g/L)			0.3 ± 0.2 d	0.2 ± 0.2 d	0.2 ± 0.0 d	0.2 ± 0.1 d	4.4 ± 0.1 a	0.7 ± 0.1 c	2.7 ± 0.2 b
Glycerol (g/L)			2.9 ± 0.1 b	2.8 ± 0.1 b	1.8 ± 0.2 c	1.9 ± 0.1 c	4.9 ± 0.1 a	4.7 ± 0.1 a	4.9 ± 0.1 a
Gluconic acid (g/L)			4.3 ± 0.1 c	4.5 ± 0.1 ab	4.4 ± 0.1 bc	4.4 ± 0.1 abc	4.0 ± 0.0 d	4.6 ± 0.1 a	4.3 ± 0.1 bc
Total polyphenols (mg/L)			2427.3 ± 42.5 d	2579.7 ± 18.0 c	2495.3 ± 2.1 cd	2548.3 ± 19.2 cd	2263.7 ± 13.2 e	2937.7 ± 93.0 b	3966.7 ± 72.0 a
Colour intensity			3.6 ± 0.0 d	4.1 ± 0.0 ab	3.7 ± 0.0 cd	3.9 ± 0.0 bc	3.7 ± 0.0 cd	4.3 ± 0.1 a	4.2 ± 0.1 a
Esters									
Hexyl acetate (µg/L)	1284	MS	7.0 ± 0.7 a	0.8 ± 0.0 b	0.8 ± 0.0 b	0.8 ± 0.0 b	1.6 ± 0.8 b	0.8 ± 0.0 b	0.9 ± 0.1 b
Isoamyl acetate (µg/L)	1120	S, MS	944.7 ± 1.8 a	12.9 ± 0.1 e	62.5 ± 10.6 c	44.2 ± 0.0 d	155.9 ± 10.0 b	4.5 ± 0.9 e	46.6 ± 7.6 cd
Phenylethyl acetate (µg/L)	1827	MS	269.8 ± 3.0 cd	45.0 ± 1.3 cd	4808.2 ± 289.1 a	2764.3 ± 20.6 b	334.0 ± 0.9 c	12.4 ± 3.6 d	120.5 ± 17.3 cd
Ethyl butyrate (µg/L)	1040	S, MS	62.6 ± 3.4 a	25.5 ± 2.0 c	21.5 ± 1.2 c	24.6 ± 0.9 c	26.3 ± 2.6 bc	31.8 ± 0.5 b	24.7 ± 1.9 c
Ethyl hexanoate (µg/L)	1242	S, MS	284.2 ± 4.0 a	15.6 ± 1.0 b	10.2 ± 1.7 c	4.4 ± 0.1 de	7.5 ± 0.9 cd	1.6 ± 0.2 e	5.3 ± 0.4 de
Ethyl octanoate (µg/L)	1445	S, MS	472.0 ± 37.9 a	32.5 ± 3.2 b	63.8 ± 5.0 b	70.3 ± 12.3 b	26.7 ± 9.5 b	34.2 ± 3.6 b	53.8 ± 3.7 b
Ethyl decanoate (µg/L)	1644	MS	153.8 ± 39.5 b	40.2 ± 3.3 c	277.3 ± 3.1 a	178.2 ± 5.0 b	56.5 ± 11.6 c	59.1 ± 8.6 c	67.9 ± 8.8 c
Ethyl dodecanoate (µg/L)	1840	MS	11.1 ± 0.6 cd	5.8 ± 0.1 d	20.6 ± 3.9 b	47.1 ± 1.7 a	9.7 ± 1.6 cd	13.2 ± 2.6 c	12.8 ± 2.3 d
Ethyl tetradecanoate (µg/L)	1998	MS	1.7 ± 0.6 d	5.2 ± 0.2 cd	18.9 ± 2.2 a	13.1 ± 0.6 b	3.7 ± 1.5 cd	6.9 ± 2.3 c	6.6 ± 1.2 c
Ethyl 4-hydroxybutyrate (µg/L)	1800	MS	7903.7 ± 449.7 a	3242.7 ± 113.8 b	1019.3 ± 13.9 cd	751.8 ± 64.5 d	3631.0 ± 170.1 b	1347.3 ± 135.0 c	1524.1 ± 4.6 c
Diethyl succinate (µg/L)	1682	MS	27.8 ± 3.2 a	7.5 ± 0.1 b	5.9 ± 0.2 b	6.0 ± 0.8 b	9.1 ± 0.2 b	7.2 ± 0.6 b	9.0 ± 0.2 b
Ethyl lactate (µg/L)	1372	MS	478.0 ± 81.6 b	243.1 ± 17.9 b	502.8 ± 17.3 b	244.1 ± 7.0 b	5852.3 ± 661.5 a	200.3 ± 13.3 b	260.6 ± 21.0 b
Diethyl malate (µg/L)	2047	MS	19.2 ± 0.8 b	12.7 ± 0.6 c	5.6 ± 0.0 e	8.4 ± 0.0 de	44.9 ± 3.1 a	9.4 ± 0.5 cd	7.9 ± 0.9 de
Ethyl vanillate (µg/L)	2653	MS	4.8 ± 0.1 bc	5.4 ± 0.3 abc	6.5 ± 0.0 a	6.2 ± 0.2 ab	3.9 ± 0.8 c	4.3 ±0.2 c	6.1 ± 1.1 ab
Ethyl cinnamate (µg/L)	2164	MS	2.5 ± 0.3 bc	2.3 ± 0.1 bc	3.4 ± 0.3 ab	4.0 ± 1.0 a	1.4 ± 0.2 c	2.7 ± 0.5 abc	2.5 ± 0.5 bc
Acids									
Butyric acid (µg/L)	1626	S, MS	162.8 ± 34.4 abc	215.2 ± 31.9 a	143.4 ± 5.1 bc	138.5 ± 12.5 bc	130.0 ± 32.7 bc	115.4 ± 1.0 c	184.9 ± 13.6 a
Isovaleric acid (µg/L)	1671	MS	176.3 ± 38.0 a	166.9 ± 15.5 ab	134.1 ± 6.6 abc	122.2 ± 10.5 bc	163.1 ± 12.0 ab	111.8 ± 1.7 c	175.2 ± 0.2 a
Hexanoic acid (µg/L)	1864	S, MS	1217.7 ± 87.0 a	177.2 ± 14.8 b	140.5 ± 3.8 bc	85.9 ± 0.6 bcd	114.1 ± 4.5 bcd	44.4 ± 8.6 d	81.3 ± 10.5 cd
Octanoic acid (µg/L)	2058	S, MS	3470.1 ± 58.3 a	254.7 ± 11.3 c	526.5 ± 1.8 b	131.3 ± 1.4 d	178.8 ± 9.1 d	41.1 ± 0.2 e	170.2 ± 19.2 d
Decanoic acid (µg/L)	2265	MS	1874.5 ± 4.9 b	121.0 ± 2.3 d	2753.3 ± 105.3 a	505.5 ± 46.6 c	400.5 ± 72.6 c	82.1 ± 15.5 d	211.5 ± 12.7 d
Dodecanoic acid (µg/L)	2482	MS	50.1 ± 5.9 bc	21.6 ± 1.1 b	86.4 ± 5.4 b	272.7 ± 45.2 a	49.5 ± 12.0 bc	33.9 ± 0.1 c	48.4 ± 6.5 bc
Tetradecanoic acid (µg/L)	2688	MS	20.9 ± 8.0 b	32.2 ± 4.0 ab	54.6 ± 11.1 a	36.6 ± 0.9 ab	29.9 ± 7.9 b	41.0 ± 15.0 ab	26.9 ± 0.9 b
Homovanillic acid (µg/L)	3099	MS	64.8 ± 6.6 a	36.7 ± 2.9 cd	46.6 ± 1.7 b	41.1 ± 1.7 bc	29.7 ± 3.7 d	30.7 ± 0.7 d	48.2 ± 0.3 b
Hydroxycinnamic acid (µg/L)	1630	MS	54.6 ± 0.8 ab	51.2 ± 0.3 cd	56.2 ± 0.3 a	53.1 ± 0.4 bc	19.0 ± 0.3 e	49.5 ± 0.5 d	53.8 ± 2.4 abc
Alcohols									
1-Hexanol (µg/L)	1375	S, MS	92.3 ± 0.8 c	93.5 ± 0.3 c	106.9 ± 1.5 b	118.7 ± 4.9 a	80.5 ± 0.3 d	74.9 ± 1.3 d	112.2 ± 1.2 b
*cis*-3-Hexen-1-ol (µg/L)	1405	MS	13.5 ± 1.2 a	11.0 ± 0.1 b	10.3 ± 0.2 b	10.7 ± 1.0 b	7.4 ± 0.4 c	6.9 ± 0.1 c	8.1 ± 0.2 c
Benzyl alcohol (µg/L)	1893	MS	10,791.6 ± 1176.7 a	11,506.5 ± 345.6 a	11,643.3 ± 79.5 a	11,741.0 ± 482.7 a	8889.2 ± 397.2 b	9011.4 ± 249.7 b	10,761.7 ± 281.6 a
3-Methylthio-1-propanol (µg/L)	1700	MS	227.0 ± 29.1 a	15.2 ± 2.5 d	36.3 ± 4.0 bcd	25.7 ± 4.6 cd	54.3 ± 2.7 bc	57.3 ± 2.4 bc	65.8 ± 4.0 b
Anisyl alcohol (µg/L)	2694	MS	70.5 ± 0.7 c	103.1 ± 2.8 ab	109.3 ± 0.9 a	109.9 ± 1.3 a	73.3 ± 5.1 c	100.3 ± 0.7 b	106.1 ± 3.6 ab
Vanillic alcohol (µg/L)	2782	MS	49.2 ± 8.1 b	69.5 ± 2.0 a	72.5 ± 0.2 a	73.3 ± 1.1 a	32.1 ± 1.9 c	56.4 ± 0.1 b	54.9 ± 7.1 b
2-Phenylethanol (mg/L)	1910	MS	12.0 ± 0.3 c	14.0 ± 0.2 b	10.2 ± 0.9 d	6.9 ± 0.3 e	11.7 ± 0.1 c	15.9 ± 0.6 a	16.2 ± 0.6 a
Phenols									
4-Vinylguaiacol (µg/L)	2260	S, MS	279.7 ± 4.3 a	55.5 ± 5.6 b	16.8 ± 2.0 c	8.8 ± 0.8 c	16.6 ± 2.6 c	11.3 ± 1.9 c	13.1 ± 4.9 c
4-Vinylphenol (µg/L)	2406	MS	1571.0 ± 106.0 a	17.0 ± 1.2 b	10.9 ± 0.6 b	5.3 ± 0.5 b	11.2 ± 2.1 b	5.2 ± 0.9 b	9.1 ± 0.5 b
Eugenol (µg/L)	1835	MS	103.2 ± 1.3 c	130.1 ± 3.9 a	131.0 ± 4.6 a	129.0 ± 1.8 a	64.5 ± 2.7 d	71.6 ± 0.5 d	115.4 ± 0.6 b
Vanillin (µg/L)	2574	MS	5.5 ± 2.2	4.9 ± 0.1	5.8 ± 0.1	7.1 ± 2.0	5.6 ± 2.0	6.1 ± 0.9	7.0 ± 0.7
Acetovanillone (µg/L)	2664	MS	26.9 ± 1.8	26.3 ± 3.3	25.1 ± 0.3	25.6 ± 0.8	24.5 ± 3.7	20.9 ± 0.2	25.6 ± 2.3
Phenol (µg/L)	2004	MS	2.0 ± 0.3 b	2.6 ± 0.1 a	2.5 ± 0.4 ab	2.9 ± 0.1 a	2.4 ± 0.1 ab	2.6 ± 0.1 a	2.6 ± 0.1 a
Lactones									
γ-Nonalactone (µg/L)	2068	S, MS	12.2 ± 0.6 b	18.4 ± 0.9 a	13.0 ± 0.0 b	13.5 ± 0.2 b	19.9 ± 0.7 a	5.3 ± 0.5 d	9.8 ± 1.4 c
γ-Butyrolactone (µg/L)	1635	MS	298.9 ± 12.9 de	310.9 ± 3.8 d	234.5 ± 0.4 e	249.0 ± 1.4 de	659.8 ± 35.3 b	816.3 ± 49.1 a	401.0 ± 8.7 c
Ethyl pyroglutamate (µg/L)	2630	MS	55.4 ± 8.0 bc	59.7 ± 5.4 bc	41.5 ± 2.5 c	40.1 ± 2.4 c	241.5 ± 23.1 a	71.4 ± 2.3 b	60.0 ± 0.4 bc
Sherry lactone 1 (µg/L)	1805	MS	16.7 ± 5.7 c	34.3 ± 1.5 ab	32.8 ± 0.1 ab	40.1 ± 3.2 a	28.8 ± 3.7 b	18.9 ± 1.7 c	29.1 ± 2.9 b
Sherry lactone 2 (µg/L)	1972	MS	79.2 ± 18.1 bc	265.7 ± 4.1 a	77.8 ± 1.0 bc	78.9 ± 4.8 bc	95.1 ± 13.6 b	82.1 ± 2.8 bc	64.8 ± 6.5 c
Aldehydes									
Phenylacetaldehyde (µg/L)	1658	MS	9.1 ± 1.6 b	19.3 ± 2.3 a	10.5 ± 2.4 b	5.7 ± 0.1 b	5.4 ± 3.7 b	8.9 ± 1.4 b	6.3 ± 1.1 b
Benzaldehyde (µg/L)	1520	S, MS	874.1 ± 17.9 a	634.5 ± 16.7 b	696.4 ± 54.6 b	686.8 ± 46.7 b	652.2 ± 35.5 b	440.3 ± 51.3 c	489.1 ± 62.5 c
Furaneol (µg/L)	1993	MS	1.8 ± 0.7 c	4.5 ± 0.9 a	3.0 ± 0.0 b	3.0 ± 0.6 b	3.6 ± 0.6 ab	2.7 ± 0.1 bc	3.6 ± 0.9 ab
Homo-furaneol (µg/L)	2067	MS	10.8 ± 2.5 b	29.4 ± 2.9 a	12.9 ± 1.3 b	11.8 ± 5.1 b	11.2 ± 0.3 b	9.3 ± 0.2 b	14.4 ± 0.4 b
Terpenes									
Linalool (µg/L)	1550	MS	8.3 ± 0.1 e	10.4 ± 0.0 c	6.7 ± 0.3 f	7.4 ± 0.1 ef	15.4 ± 0.3 a	13.2 ± 0.2 b	9.6 ± 0.6 d
α−Terpineol (µg/L)	1705	MS	2.9 ± 0.1 cd	3.9 ± 0.8 bc	2.2 ± 0.1 d	3.1 ± 0.2 cd	6.9 ± 0.4 a	4.6 ± 0.6 b	4.5 ±0.2 b

^1^ Retention index. ^2^ ID, identification method: S, mass spectra and retention index in agreement with that of the authentic compound under similar GC-MS conditions; MS, mass spectra in agreement with the spectra database reported in the literature. Different letters indicate significant differences for *p* < 0.05.

## Data Availability

The original contributions presented in the study are included in the article, further inquiries can be directed to the corresponding author. The data are not publicly available due to privacy restrictions.

## Supplementary Materials

The following supporting information can be downloaded at: https://www.mdpi.com/article/10.3390/foods13152455/s1, Figure S1: Experimental design used in the study; Table S1: List of 21 strains used in this study with related GenBank accession number of D1/D2 sequence region; Figure S2: Kinetics of CO_2_ production (% *w/v*) during the fermentation of cherry must B by six selected yeasts and *S. cerevisiae* EC1118^®^ (control); Figure S3: Total amount of acetate esters (blue columns), fatty acid ethyl esters (red columns), and fatty acids (green columns) detected in cherry-based wines; Table S2: Compounds detected above their respective thresholds and relative OAV of cherry-based wines; Table S3: Results of AHC for cherry-based wines and aroma compounds; Figure S4: PLS-DA plot of the two components (t1 and t2) obtained by analyzing aroma compounds of cherry-based wines; Figure S5: PCA plot of the two factors (F1 and F2) obtained by analyzing all compounds of cherry-based wines; Table S4: Loading factors of compounds analyzed in cherry-based wines obtained by PCA.
