# Supplementary material for: Fermentation Performances and Aroma Contributions of Selected Non-Saccharomyces Yeasts for Cherry Wine Production
Source: Foods. 2024 Aug 3;13(15):2455. doi: 10.3390/foods13152455 (PMC11312165; doi:10.3390/foods13152455)
Supplement: Supplementary file 1 [file foods-13-02455-s001.zip › foods-3110094-supplementary.pdf]

SUPPLEMENTARY MATERIALS

Figure S1. Experimental design used in the study.

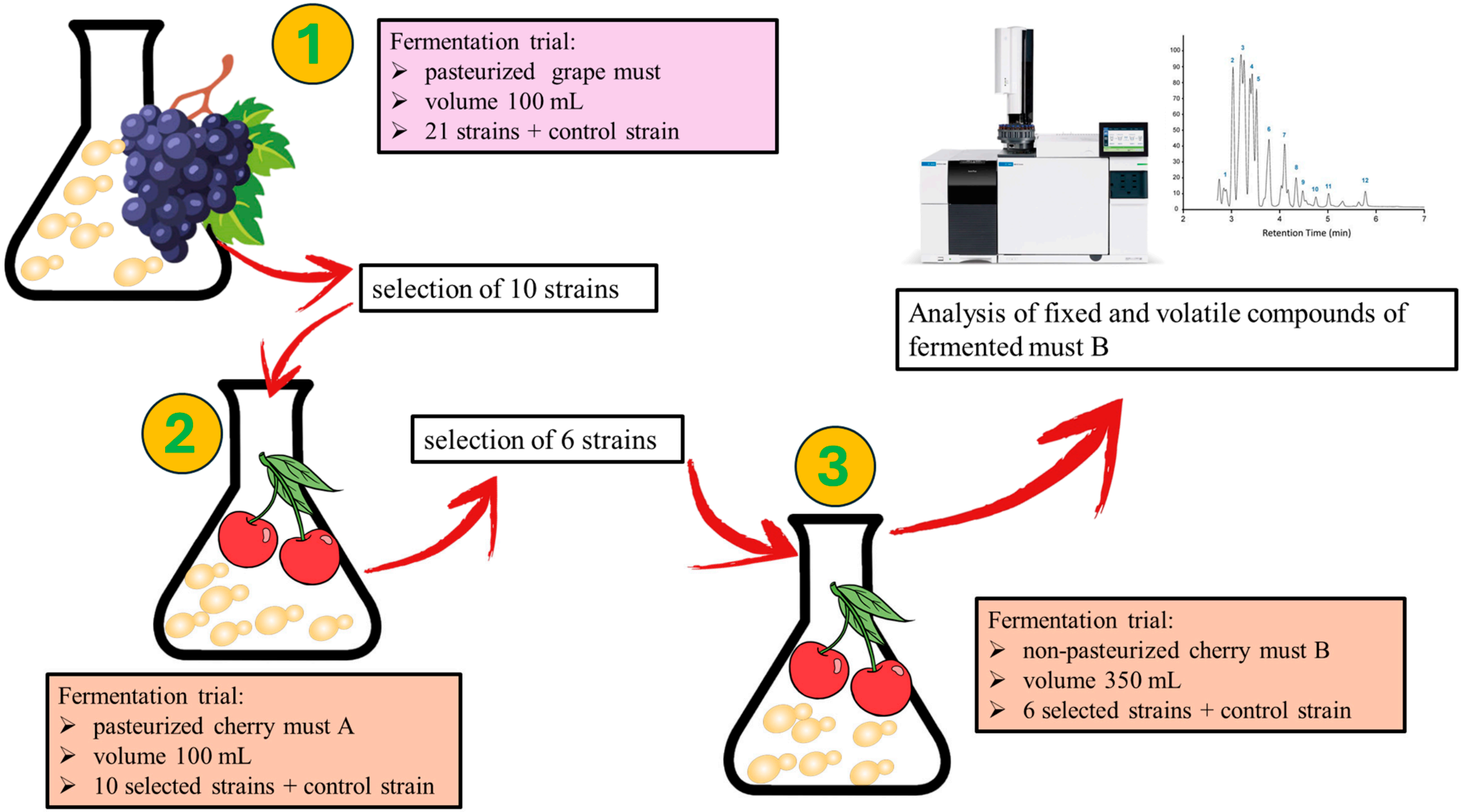

Table S1. List of 21 strains used in this study with related Genbank accession number of D1/D2 sequence region.

Species	strain	source	accession number	reference
Hanseniaspora meyeri	Y1A	apple juice	MG478470	Lorenzini et al. 2018
Hanseniaspora osmophila	Ho3	grape	PP107913	Lorenzini and Zapparoli, 2019
Hanseniaspora osmophila	Ho22	grape	PP107914	Lorenzini and Zapparoli, 2019
Hanseniaspora pseudoguilliermondii	YR6	apple juice	MG478474	Lorenzini et al. 2018
Hanseniaspora vineae	Hv1	grape	PP107915	Lorenzini and Zapparoli, 2019
Hanseniaspora vineae	Hv20	grape	PP107916	Lorenzini and Zapparoli, 2019
Hanseniaspora vineae	Hv45	grape	PP107917	Lorenzini and Zapparoli, 2019
Hanseniaspora valbiensis	Y2D	apple juice	MG478468	Lorenzini et al. 2018
Lachancea fermentati	Lf2	olive oil	PP107918	this study
Lachancea lanzarotesis	Ll5	grape	PP107919	Lorenzini and Zapparoli, 2019
Lachancea lanzarotesis	Ll7	grape	PP107920	Lorenzini and Zapparoli, 2019
Starmerella bacillaris	Stb3	grape	PP107921	Lorenzini and Zapparoli, 2019
Starmerella bacillaris	Stb7	grape	PP107922	Lorenzini and Zapparoli, 2019
Starmerella bacillaris	Stb34	grape	PP107923	Lorenzini and Zapparoli, 2019
Starmerella bacillaris	Stb142	grape	PP107924	Lorenzini and Zapparoli, 2019
Starmerella bacillaris	Stb91	grape	PP107925	Lorenzini and Zapparoli, 2019
Torulaspora delbrueckii	Tb7	grape	PP107926	Lorenzini and Zapparoli, 2019
Wickerhamomyces anomalus	Wa847	wine	PP107939	this study
Zygosaccharomyces bailii	Zb17	grape	PP107927	Lorenzini and Zapparoli, 2019
Zygosaccharomyces bailii	Zb19	grape	PP107928	Lorenzini and Zapparoli, 2019
Zygosaccharomyces bailii	Zb23	vinegar	PP107938	this study

Figure S2. Kinetics of CO_2 production (% w/v) during the fermentation of cherry must B by six selected yeasts and *S. cerevisiae* EC1118® (control).

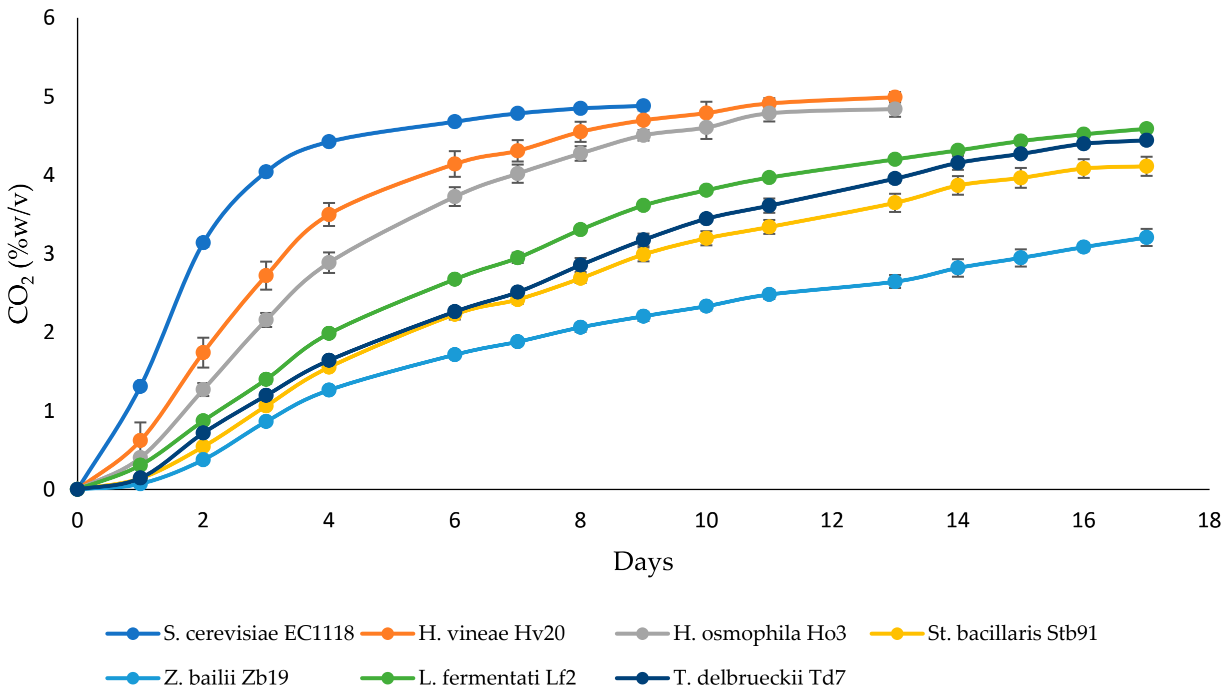

Figure S3. Total amount of acetate esters (blue columns), fatty acid ethyl esters (red columns) and fatty acids (green columns) detected in cherry-based wines fermented by *S. cerevisiae* EC1118® (control), *T. delbrueckii* Td7 (TD), *H. vineae* Hv20 (HV), *H. osmophila* Ho3 (HO), *L. fermentati* Lf2 (LF), *St. bacillaris* Stb91 (STB) and *Z. bailii* Zb19 (ZB).

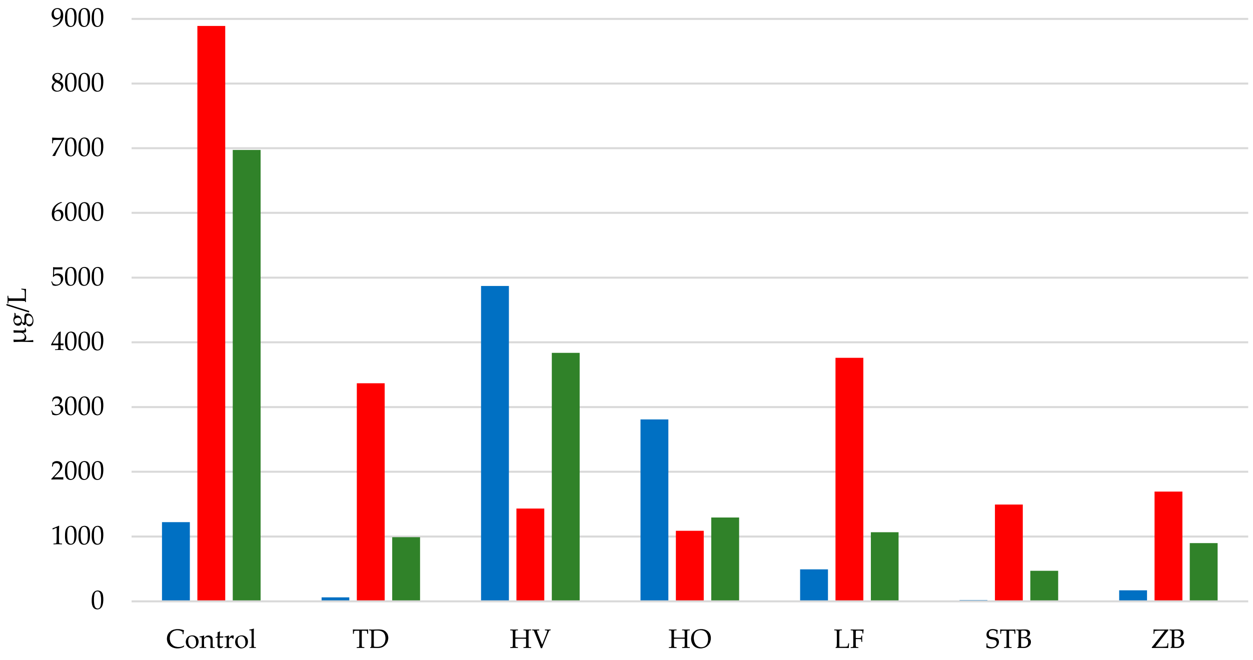

Table S2. Compounds detected above their respective thresholds and relative OAV of cherry-based wines produced by *S. cerevisiae* EC1118® (control), *T. delbrueckii* Td7 (TD), *H. vineae* Hv20 (HV), *H. osmophila* Ho3 (HO), *L. fermentati* Lf2 (LF), *St. bacillaris* Stb91 (STB) and *Z. bailii* Zb19 (ZB). Values are mean with standard deviation of three independent trials.

	**odour threshold (μg/L)[1]**	**descriptors**	**OAV**						
			**control**	**TD**	**HV**	**HO**	**LF**	**STB**	**ZB**
Isoamyl acetate	30	banana	31.5	0.4	2.1	1.5	5.2	0.2	1.6
Phenylethyl acetate	250	rosa, floreal	1.1	0.2	19.2	11.1	1.3	0.0	0.5
Ethyl butyrate	20	fruity	3.1	1.3	1.1	1.2	1.3	1.6	1.2
Ethyl hexanoate	14	fruity, anise	20.3	1.1	0.7	0.3	0.5	0.1	0.4
Ethyl decanoate	200	caramel, fruity	0.8	0.2	1.4	0.9	0.3	0.3	0.3
Ethyl cinnamate	1.1	cinnamon, balsamic	2.3	2.1	3.1	3.6	1.3	2.5	2.2
2-Phenylethyl alcohol	14	floreal, rose	0.9	1.0	0.7	0.5	0.8	1.1	1.2
4-Vinylguaiacol	40	spice, curry	7.0	1.4	0.4	0.2	0.4	0.3	0.3
4-Vinylphenol	180	phenolic, medicinal	8.7	0.1	0.1	0.0	0.1	0.0	0.1
Eugenol	6	clove, spice	17.2	21.7	21.8	21.5	10.8	11.9	19.2
Phenylacetaldehyde	1	floreal, honey	9.1	19.3	10.5	5.7	5.4	8.9	6.3
Butyric acid	173	cheese	0.9	1.2	0.8	0.8	0.8	0.7	1.1
Isovaleric acid	33	candy, cheese	5.3	5.1	4.1	3.7	4.9	3.4	5.3
Hexanoic acid	420	cheese, greasy	2.9	0.4	0.3	0.2	0.3	0.1	0.2
Octanoic acid	500	cheese, rancid	6.9	0.5	1.1	0.3	0.4	0.1	0.3
Decanoic acid	1000	rancid fat	1.9	0.1	2.8	0.5	0.4	0.1	0.2

[1]Escudero A, Campo E, Fariña L, *et al.* Analytical characterization of the aroma of five premium red wines. Insights into the role of odor families and the concept of fruitiness of wines *J. Agric. Food Chem.* 2007;**55**:4501–4510.

1

Table S3. Results of AHC for cherry-based wines and aroma compounds. Values indicating the optimal selection of clusters for AHC are in bold.

	cherry-based wines				aroma compounds			
**results by class**								
number of clusters	2	3	4	5	2	3	4	5
Hartigan index (H)	2.686	2.100	1.801	1.479	24.312	12.692	6.630	5.870
H(k-1) - H(k)	0.013	**0.586**	0.300	0.321	−5.614	**11.620**	6.062	0.760
**results by cluster**								
clusters	W1	W3	W2		C2	C3	C1	
Silhouette scores	-	0.124	0.429		0.643	0.324	0.163	

Figure S4. PLS-DA plot of the two components (t1 and t2) obtained by analyzing aroma compounds of cherry-based wines produced by *S. cerevisiae* EC1118® (control), *T. delbrueckii* Td7 (TD), *H. vineae* Hv20 (HV), *H. osmophila* Ho3 (HO), *L. fermentati* Lf2 (LF), *St. bacillaris* Stb91 (STB) and *Z. bailii* Zb19 (ZB).

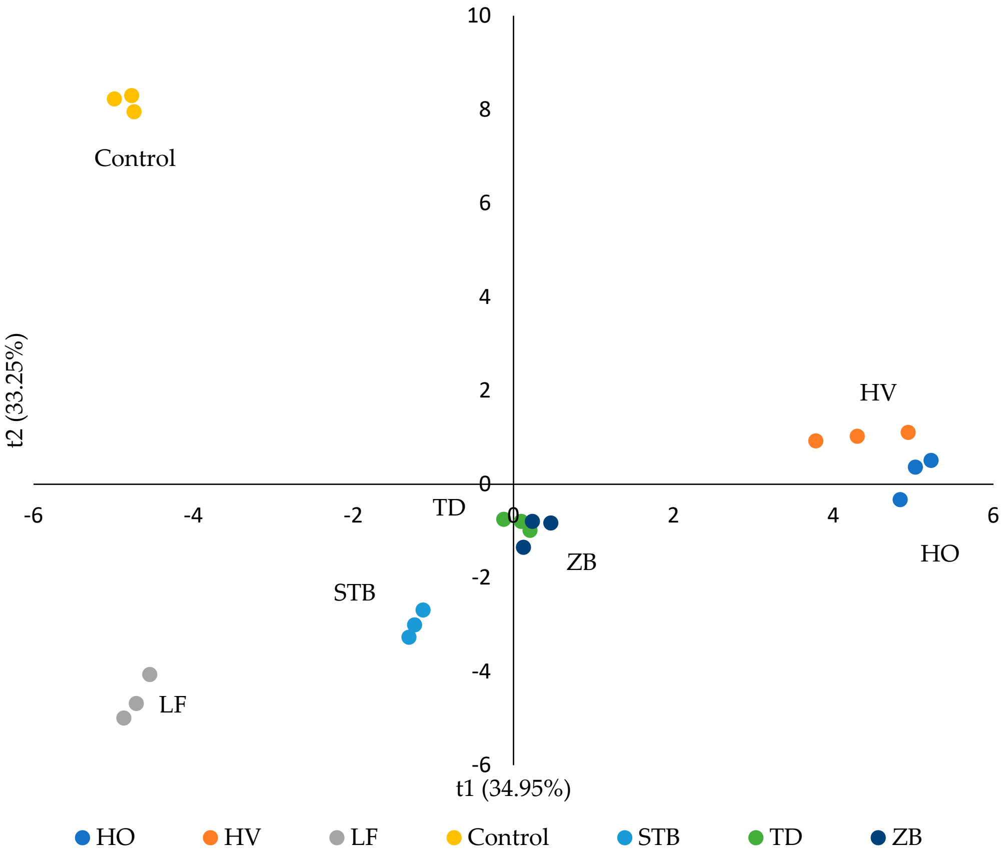

Figure S5. PCA plot of the two factors (F1 and F2) obtained by analyzing all compounds of cherry-based wines produced by *S. cerevisiae* EC1118® (control), *T. delbrueckii* Td7 (TD), *H. vineae* Hv20 (HV), *H. osmophila* Ho3 (HO), *L. fermentati* Lf2 (LF), *St. bacillaris* Stb91 (STB) and *Z. bailii* Zb19 (ZB).

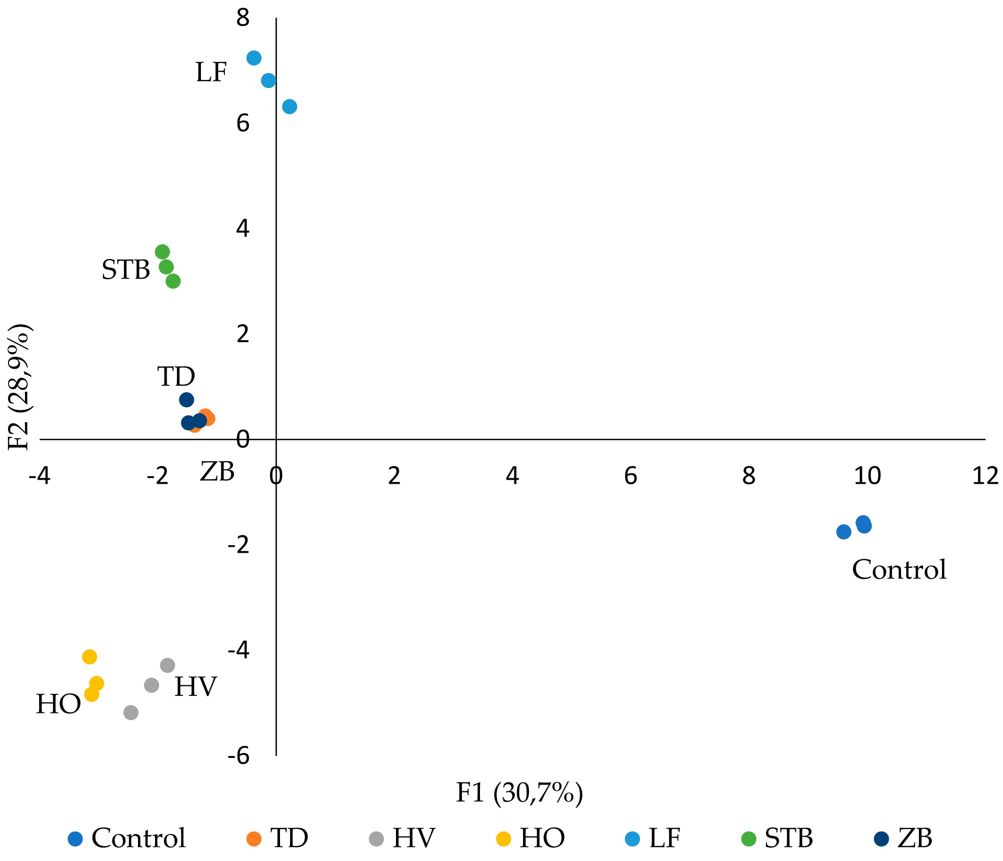

Table S4. Loading factors of compounds analysed in cherry-based wines obtained by PCA with values <− 0.800 or > 0.800 at least in one of the two F1 and F2.

	**F1**	**F2**
Glycerol	-0.424	-0.805
Hexyl acetate	0.937	-0.290
Isoamyl acetate	0.950	-0.271
Ethyl butyrate	0.880	-0.311
Ethyl hexanoate	0.966	-0.198
Ethyl octanoate	0.966	-0.113
Ethyl tetradecanoate	-0.270	0.825
Ethyl 4-hydroxybutyrate	0.843	-0.500
Diethyl succinate	0.905	-0.334
Ethyl vanillate	-0.019	0.806
Etyl cinnamate	0.045	0.835
cis-3-Hexen-1-ol	0.840	0.375
3-Methylthio-1-propanol	0.867	-0.333
Vanillic alcohol	-0.086	0.909
Linalool	-0.447	-0.854
α−Terpineol	-0.470	-0.821
4-Vinylguaiacol	0.961	-0.209
4-Vinylphenol	0.958	-0.203
Eugenol	0.166	0.843
Benzaldehyde	0.834	0.123
Hexanoic acid	0.972	-0.185
Octanoic acid	0.979	-0.145
Homovanillic acid	0.831	0.233
